# Diversity Enhances NPP, N Retention, and Soil Microbial Diversity in Experimental Urban Grassland Assemblages

**DOI:** 10.1371/journal.pone.0155986

**Published:** 2016-05-31

**Authors:** Grant L. Thompson, Jenny Kao-Kniffin

**Affiliations:** Section of Horticulture, School of Integrative Plant Science, Cornell University, Ithaca, New York, United States of America; Institute of Tibetan Plateau Research, CHINA

## Abstract

Urban grasslands, landscapes dominated by turfgrasses for aesthetic or recreational groundcovers, are rapidly expanding in the United States and globally. These managed ecosystems are often less diverse than the natural or agricultural lands they replace, leading to potential losses in ecosystem functioning. Research in non-urban systems has provided evidence for increases in multiple ecosystem functions associated with greater plant diversity. To test if biodiversity-ecosystem function findings are applicable to urban grasslands, we examined the effect of plant species and genotypic diversity on three ecosystem functions, using grassland assemblages of increasing diversity that were grown within a controlled environment facility. We found positive effects of plant diversity on reduced nitrate leaching and plant productivity. Soil microbial diversity (Mean Shannon Diversity, *H’*) of bacteria and fungi were also enhanced in multi-species plantings, suggesting that moderate increments in plant diversity influence the composition of soil biota. The results from this study indicate that plant diversity impacts multiple functions that are important in urban ecosystems; therefore, further tests of urban grassland biodiversity should be examined *in situ* to determine the feasibility of manipulating plant diversity as an explicit landscape design and function trait.

## Introduction

Turfgrasses are the defining vegetation in urban landscapes, including residential lawns, institutional grounds, municipal parks, recreational fields, golf courses, and civic greens, and because of their connectivity across property lines, can be considered a new ecosystem type—the urban grassland [1]. The extent of these grasslands has expanded rapidly in the United States in recent decades, as the population becomes more urbanized [2, 3]. During the recent decadal census, the U.S. Census Bureau [4] found that over 80% of the nation lives in urban areas—an increase of about 1.8% over the previous decade. Furthermore urbanization trends are increasing even more rapidly in certain regions. Jantz [5] found from 1990–2000 there was a 61% increase in urbanized areas in the Chesapeake Bay watershed. The expansion of urban grasslands into natural, pastoral, and agricultural ecosystems brings about large shifts in vegetative cover from multi-species ecosystems to monotypic stands of turfgrasses. Turfgrasses are estimated to cover up to 2% of the total U.S. terrestrial land area, which is an area three times greater than any irrigated crop [6]. In fact, regionally, turfgrass cover can reach very high proportions of the urban landscape, including up to 23% of the Columbus, Ohio metropolitan area [7]. The shift in plant species composition and overall loss of plant diversity when urban grasslands are created, is likely to alter many ecosystem functions [8].

Urban grasslands contain relatively few species compared to the landscapes they replace [9], which could have negative impacts on many important functions. As a land-use type, they are frequently identified as contributing to declining ecosystem services, such as being sources of non-point source pollution, reduced biodiversity, and increased greenhouse gas emissions [10–14]. Further effects of urban grasslands include intensive land management practices that may adversely affect, or at least alter, soil biological diversity and function, as compared to landscapes with greater diversity [15, 16]. Since many biogeochemical cycles are mediated by microorganisms, impacts on soil microbial communities could further lead to the degradation of many ecosystem traits. Several researchers have reported on the effects of urban grasslands on terrestrial biogeochemical cycling, particularly involving carbon and nitrogen [6, 8, 17–19].

Although the typical turfgrass landscape is species-poor, there is potential to enhance diversity and ecosystem multi-functionality in these urban grasslands by applying ecological theory, derived from the biodiversity-ecosystem function (BEF) literature, to their design. Substantial theoretical and experimental work has occurred over the last few decades regarding the functional outcomes of biodiversity in ecosystems [20–23]. Experimental and observational studies of short and tall grass prairies (native grasslands), drylands, forests, agricultural, and aquatic ecosystems represent the majority of biodiversity and ecosystem functioning (BEF) research [20, 22–28]. Greater species richness has been shown to increase productivity, as greater diversity in plant physiological traits has been linked to a more complete resource utilization within an ecosystem [29, 30]. However, abiotic controls ultimately limit the potential productivity of the ecosystem, producing an asymptotic response [22, 31]. Decreased nitrate leaching is an outcome of greater resource utilization in diverse communities, and is relevant to urban ecosystem quality [32–34].

To date, biodiversity effects have not been well studied in urban grasslands [9], yet findings from BEF research suggest increasing biodiversity in urban grasslands has the potential to address many of the common environmental issues associated with these ecosystems, particularly N retention. To determine if BEF theory can be applied to urban grassland systems, we conducted an experimental manipulation of species and genotypic diversity in grassland mesocosm assemblages at a controlled environment facility, to measure the effects of increasing plant diversity on nitrate leaching, plant productivity, and soil microbial diversity. Based on the literature we summarized above, we hypothesized that increasing plant species and genotype diversity in urban grasslands would i) increase plant productivity, ii) decrease nitrate leaching, and iii) be associated with greater belowground diversity in soil microbial communities.

## Materials and Methods

### Grassland assemblages and maintenance

The plants, consisting of 8 species and 7 genotypes, were chosen to represent cool-season North American turfgrasses found in urban grasslands and available from commercial vendors (Table 1). Modern turfgrass breeding lines show significant polymorphisms within a species [35] that contribute to plasticity of grass form and function. To account for this genetic and functional variation within a species, genotypic richness was added to the grassland assemblages. For example, *Festuca rubra* var. commutata 'Intrigue 2' produces an allelochemical from the roots (meta-Tyrosine) that inhibits growth of other species [36], while another genotype, *Festuca rubra* var. commutata 'Zodiac', does not. Thus, genotypes of crop species may show high trait variation within a species than between species, when measuring impacts on ecosystem function.

**Table 1:** Full list of plant species and genotypes comprising the grassland assemblages.

Botanical Name	Common Name	Abbreviation
*Festuca arundinacea* 'Bullseye'	Tall Fescue	TFBE
*Festuca arundinacea* 'Falcon V'	Tall Fescue	TFF5
*Festuca ovina* var. duriuscula 'Spartan II'	Hard Fescue	HFS2
*Festuca rubra* 'Garnet'	Creeping Red Fescue	CRFG
*Festuca rubra* var. commutata 'Zodiac'	Chewings Fescue	CFZO
*Festuca rubra* var. commutata 'Intrigue 2'	Chewings Fescue	CFI2
*Lolium perenne* 'Amazing GS'	Perennial Rye Grass	PRGA
*Lolium perenne 'Fiesta 4'*	Perennial Rye Grass	PRGF
*Poa annua var*. *reptans 'Two Putt'*	Annual Bluegrass	ABTP
*Poa pratensis 'Bedazzled'*	Kentucky Bluegrass	KBGB
*Poa supina 'Supranova'*	Supina Bluegrass	SBGS
*Trifolium repens 'Microgreen'*	Microclover	MCMG

The assemblages were comprised of 1-, 3-, 6-, and 12-components of plants on the list, selected at random. Each of the four component assemblages were replicated.

The 13 grassland assemblages, consisting of six monocultures, three 3-component, three 6-component, and one 12-component polycultures were grown in mesocosms with five replicates each (total n = 65). The specific composition of the grassland assemblages were selected at random, using a random number generator in Microsoft Excel, from the possible pool of 12 components. For example, to determine the composition of the 6-component assemblages, the full list of 12 components were included in each of three columns (ie. n = 3 replicates) and random numbers were used to rearrange the list by ascending number order, thereby facilitating selection of the top six components in each column (ie. 6-component assemblage replicate). Each assemblage is described in Table 2. The 12-component system is comprised of eight plant species, of which there are additional representatives of genotypes within a species exhibiting phenotypic diversity. For example, the “fine fescues” include species, subspecies, and genotypes that are well known for displaying phenotypic variation.

**Table 2:** Composition of the grassland assemblages consisting of 1-, 3-, 6-, and 12-component systems.

Diversity Level	Trt. ID	Component composition					
Monoculture	1	PRGA	-	-	-	-	-
	2	SBGS	-	-	-	-	-
	3	CRFG	-	-	-	-	-
	4	KBGB	-	-	-	-	-
	5	MCMG	-	-	-	-	-
	6	TFF5	-	-	-	-	-
3-Poly.	7	TFBE	KBGB	MCMG	-	-	-
	8	HFS2	CFZO	ABTP	-	-	-
	9	TFF5	SBGS	MCMG	-	-	-
6-Poly.	10	TFF5	HFS2	CFZO	CFI2	ABTP	MCMG
	11	CFI2	PRGA	PRGF	KBGB	SBGS	MCMG
	12	TFBE	TFF5	HFS2	CFZO	PRGA	PRGF
12-Poly.	13	All 12					

Each of 13 assemblages (indicated by Treatment ID number) was replicated in five mesocosms (total = 65 mesocosms). The grassland assemblages consisted of six different 1-way component monocultures (treatments 1–6), three 3-component polycultures (treatments 7–9), three 6-component polycultures (treatments 10–12), and a 12-component polyculture (treatment 13). See Table 1 for full botanical and common names of component abbreviations used.

The mesocosm containers measured 30l x 38w x 18d cm, and were fitted with custom internal lysimeters to facilitate leachate collection. Mesocosms were filled to within 5 ± 0.5 cm from the top with a soil mixture and watered until the soil settled. The soil used in the mesocosms was a mixture of sand and topsoil at a 2:1 ratio. The topsoil was derived from Genoa, New York and sand was sourced from the eastern shore of Maryland and consisted primarily of medium and coarse particle size (0.25 to 0.5 mm) with less than 1.5% silt or clay. The soil mixture was homogenized using a concrete mixer (Stone Construction Equipment Inc. Model 950MP, Honeoye, NY). Soil and sand were blended for approximately 5 min or until a homogeneous mix was achieved. The blended growing media pH was 8.0.

Polyculture seed mixes were blended evenly on a weight basis. Fifty seeds of each type were counted and weighed four times to determine an average seed weight. Seeding rates were calculated to achieve approximately 3,600 seeds per mesocosm (approximately 32,000 seeds m^-2^ representing a typical average seeding density for turfgrasses). Prior to seeding, *Trifolium repens* ‘Microgreen’ was inoculated with D-Nure (INTX Microbials, LLC, Kentland, IN) following the inoculant manufacturer’s instructions. Hereafter, the term grass or turfgrass includes *T*. *repens* (a broadleaf legume), unless otherwise stated.

In October 2012, the mesocosms containing *P*. *pratensis* and *P*. *annua* were hand seeded. After bi-directional seeding for even dispersion, seeds were lightly pressed to ensure adequate seed-soil contact. Ten days later, the remaining grassland seeds were added to all mesocosms. After four weeks total, greater than 50% of mesocosms showed over 50% germination.

Grassland assemblages were grown in mesocosms for seven months at the Cornell University Kenneth Post Labs controlled environment greenhouse facility (Ithaca, New York). Room temperature and supplemental lighting were regulated via an Argus Control System (White Rock, British Columbia, Canada) with automated controller sensors suspended above the mesocosms. Environmental conditions including temperature, relative humidity, ambient CO_2_, and light conditions are reported in Table 3. An array of twenty PL2000 400w HPS lamps by PL Light Systems were used to deliver supplemental lighting daily for 16 h.

**Table 3:** Greenhouse environmental parameters.

	Temp.°C	% Rel. Humidity	CO_2_ ppm	PAR μmol m^-2^ s^-1^
Minimum	12.9	9.4	241.0	0.0
Maximum	31.5	83.2	687.0	1926.0
Average	21.3	44.3	362.6	259.7

Temperature, relative humidity, CO_2_ and photosynthetically active radiation were automatically logged at 15-minute intervals continuously for the duration of the experiment.

Mesocosms were hand watered with tap water as needed, averaging approximately 2–3 L wk^-1^, depending on growing conditions. Liquid 21:5:20 (N-P-K) fertilizer was applied at a rate of 200ppm-N from a bulk tank. Fertilizer concentrate was diluted via a Dosatron D14MZ2 14GPM injector by Dosatron International (Clearwater, FL). Fertilizer was applied when growth rates slowed or turf appeared stressed, approximately 1 L every two weeks. Approximately 2.5 g N per mesocosm was added during the course of the experiment, which corresponds to a high-end recommendation for Kentucky bluegrass fertilization of approximately 200 kg N ha^-1^.

### N leaching measurement

Leachate was collected twice during the experiment. Data for the second leaching are reported here. Leaching was conducted by adding 5L of water to mesocosms to saturate the soil and cause slight pooling on the soil surface. Lysimeter collection tubes were plugged to allow water to equilibrate with soil pores for 1 h. Mesocosms were allowed to drain for 1 h and 50 ml leachate samples were collected. Samples were filtered and stored at –20°C after collection. For leachate analysis, samples were thawed at 4°C overnight and run on an AQ2 Discrete Analyzer by Seal Analytical (Mequon, WI, USA).

### Productivity measurement

Clipping collection began six weeks after initial seeding and continued weekly for seventeen weeks, ending in mid-March 2013. Grasses were clipped at a height of 6.5 ± 0.5 cm and clippings were placed in labeled paper envelopes and dried for 72 h at 50°C. Samples were weighed directly from the drying oven.

Twenty-three weeks after initiation, one quarter (15 x 19 cm) of each mesocosm was destructively harvested for analyses of standing above and belowground biomass. Grasses were cut at the soil surface and samples were dried following the clipping protocol. See below for rhizosphere soil sampling protocols. Root masses, bulk soil, and unsampled rhizosphere soil were placed in labeled plastic bags and stored at 9°C until the samples could be washed. Roots were isolated by washing samples with tap water and sequential sieving through 4.75 mm (No. 4) and 2 mm (No. 10) standard testing sieves (Advantech, ASTM E-11, USA). Materials passing a 2 mm sieve were discarded. Root samples were dried at 50°C for 72 h and weighed. Root samples were collected within 7 d of harvest.

One-quarter (15 x 19 cm) subsample of each mesocosm was destructively harvested to characterize grassland composition. Root-bound soil and aboveground biomass were kept intact to facilitate identification. Grasses were carefully separated from the soil mass to keep crowns, tillers, rhizomes, stolons, and roots intact. Grasses were visually sorted into up to seven categories; cultivars of the same species and all fine fescues were pooled, respectively (Table 1).

Once sorted, grasses were counted by crown to determine the number of individuals present in the harvested quarter. Daughter plants connected to parent plants by rhizomes or stolons were counted as single individuals. After polycultures were sorted and counted shoots were separated from roots. All materials were dried at 50°C for 72 h and weighed. Sorted aboveground biomass C and N content were determined using a LECO CN-2000 combustion analyzer (LECO Corporation, St. Joseph, MI).

### Microbial community composition

Rhizosphere soil is operationally defined by this study as the soil closely bound to roots, which can be freed by mechanical manipulation of the root mass after loose soil has been removed. Soils and root masses from the first destructive harvest were shaken for 60 s to free bulk soil. Careful manipulation of soils remaining in the root masses freed rhizosphere soil. Two subsamples (~50 g) of rhizosphere soil were collected from each mesocosm. Root masses, bulk soil, and remaining rhizosphere soil were saved for belowground biomass measurements. Rhizosphere soil samples were lyophilized with a Labconco FreeZone 2.5L Benchtop system (Kansas City, MO) and stored at –20°C until DNA extraction. DNA extraction, amplification, and T-RFLP. Rhizosphere soil samples were mechanically ground using 13/16 in cylindrical burundum grinding media (EA Advanced Ceramics, E. Palestine, OH). The MoBio Power Soil DNA Isolation Kit by MoBio Laboratories, Inc. (Carlsbad, CA) was used for extraction of the ground soils. Bacterial 16S rRNA genes were amplified for terminal restriction fragment length polymorphism (T-RFLP) using Bac8F* (5-AGAGTTTGATCCTGGCTCAG-3) with the 5’ end 6-FAM labeled and unlabeled 1492R (5’-CGGTTACCTTGTTACGACTT-3) universal primers. Fungal 18s rRNA genes were amplified using universal fungal primers LROR* (5-ACCCGCTGAACTTAAGC-3) and LR5 (5 TCCTGAGGGAAACTTCG-3). Each 50 μC reaction contained 1.5 μr labeled forward primer, 0.5 μl unlabeled reverse primer, 10 μu Go Taq Buffer, 2.5 mM magnesium chloride, and 2 μm template DNA. Reactions were cycled at 95°C for 3 min, cycled 35 times at 95°C for 30 s, 50°C for 30 s, and 72°C for 45 s, and a 12 min final extension at 72°C [37]. Reactions were carried out in duplicate, then pooled.

PCR products were cleaned, concentrated, and desalted via Qiaex II Gel Extraction Kit by Qiagen Technologies (Germantown, MD) with repeated elution to increase yield. DNA concentration was quantified by using Quant-iT PicoGreen dsDNA Kit by Invitrogen (Carlsbad, CA) using a 96-well BioTek Synergy HT luminescence microplate reader with Gen5 software for data collection and analysis (Winooski, VT). Cleaned PCR products were normalized to achieve a DNA template target concentration of ~350 ng per 15 μl reaction for the enzymatic digest. Digestion reactions were carried out using the *Hae*III restriction enzyme with CutSmart Buffer. Cleaned digests were dried in a rotary evaporator and resuspended in 9.7μl formamide and 0.3 μl of 500 LIZ size standard (Applied Biosystems). Terminal restriction fragments (TRFs) were quantified with a 3730XL gas capillary auto analyzer (Applied Biosystems) following the EPA-114-A Rev. 8 method. See Liu [38] for more details on T-RFLP characterization of microbial communities.

### Statistical Analysis

To test the effect of diversity-level treatments on response variables, ANOVA was run using JMP Pro 10 (SAS, Cary, NC). Data transformations were performed as necessary to fit model assumptions of normality and constant variance. Tukey’s Honestly Significant Difference (HSD) tests were used to determine diversity effects (p <0.05). T-RFLP data analysis are based on published protocols Berthrong [37], Abdo [39], with modifications noted below. Electropherograms were analyzed and compared to size standards using Peak Scanner ver. 1.0 (Applied Biosystems). Bacterial peaks were analyzed within the standard (50–800 bp) and above 50 units of height. Fungal peaks outside of the standard and below 200 units of height were excluded, to meet data input parameters of PC-Ord software (ver. 5.31). The online T-Rex software filtered noise, clustered, and aligned peaks in order to determine the TRFs present. TRF peak area was averaged over two laboratory replicates and relitivedized within samples for the bacterail and fungal samples separately. TRFs not appearing in greater than 3 samples were omitted. PC-Ord was utilized to perform non-metric multidimensional scaling (NSM) to visualize similarities within the TRF data. A squareroot transformation was applied to both the bacterial and fungal data to reduce apparent variability for analysis. Outliers exhibiting greater than two times the standard deviation of the respective distance measures were detected in both the bacterial and fungal analyses. Four outliers were removed from bacterial analyses as the samples were not correlated with specific treatments. Three of five outliers for fungal analyses were related to the monoculture *Trifolium* treatment, thus ouliers were included in the analyses. The Shannon indices (*H’*) of soil TRF diversity for bacterial and fungal communities were calculated using the online EstimateS software (Robert K Colwell software, Stoors, CT). Data was scaled one hundred-fold to produce integer abundance values for *H’* calculations.

## Results

### Nitrate leaching

**Fig 1:**
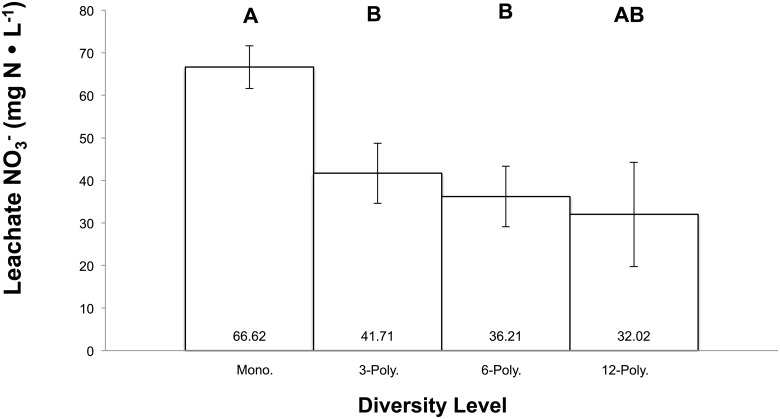
Mesocosm leachate NO_3_^-^ (mg N ∙ L^-1^) concentration by diversity level. Leached NO_3_^-^ values are reported by grassland diversity levels: Monocultures or 1-way component (mean of treatments 1–6), three 3-component polycultures (mean of treatments 7–9), three 6-component polycultures (mean of treatments 10–12), and a 12-component polyculture. Error bars represent ±1 SEM. Bars with contrasting letters indicate significantly different means by Tukey’s HSD test.

Nitrate (NO_3_^-^) concentrations (mg L^-1^) in leachate were analyzed by plant diversity levels. These included the 1-component, 3-component, 6-component, and 12-component grasslands (Fig 1). Nitrate levels diminished significantly in the 3- and 6-component assemblages compared to the monoculture (*P* = 0.0028). The 12-component group showed a similar trend in reduced NO_3_^-^ leaching, but the low replication (n = 5) did not allow for statistical differentiation from monoculture treatments (Fig 1).

Within a diversity level, we compared nitrate levels to assess the effect of a dominant species on N dynamics (S1 Fig). Of the monocultures, *Festuca arundinacea* (tall fescue, Falcon V genotype) resulted in the lowest levels of leached nitrate, while *Trifolium repens* (Microgreen clover) had the highest 5.64 ± 0.69 and 10.70 ± 0.69 mg L^-1^, respectively. Polyculture treatments containing tall fescue, Falcon V (mesocosms 12 and 13) were significantly lower than other polyculture treatments. However, when the clover was included with tall fescue Falcon V in mesocosms 9 and 10, nitrate leaching increased and was not significantly different from other polyculture treatments. Furthermore, the lowest overall nitrate leaching rates (4.90 ± 0.69 mg L^-1^) was observed in treatment 8 of the 3-component assemblage.

### Total aboveground productivity increases with diversity

Total aboveground productivity consisting of 17 weeks of leaf clippings and final standing aboveground biomass was positively associated with diversity level and significantly different between monoculture treatments and 3-and 6-component polycultures (*P* = 0.0059, R^2^ = 0.18). When clippings and standing biomass were analyzed separately, we found that the harvested biomass of leaf clippings over 17 weeks did not indicate significant differences among treatments grouped by diversity-level (*P* = 0.13, R^2^ = 0.09). Standing aboveground biomass in the 3-component polycultures was significantly higher from monoculture treatments, however 6- and 12-component polycultures were not distinguishable from either monocultures or 3-part polycultures (*P* = 0.0038*, R^2^ = 0.20). Belowground biomass did not yield significant differences at any diversity treatment level (*P* = 0.81, R^2^ = 0.02). The low replication number of the 12-component polycultures (n > 5) contributed to the high variability in community productivity, making it difficult to distinguish differences between monoculture and polyculture treatments (Table 4).

**Table 4:** The productivity (g•m^-2^ dry weight) of grassland assemblages.

Productivity	Mono.		3-Poly.		6-Poly.		12-Poly.	
Leaf clippings	598.19 ± 9.628	*A*	613.25 ± 13.62	*A*	631.63 ± 13.62	*A*	693.51 ± 23.59	*A*
Aboveground	530.12 ± 27.02	*B*	677.04 ± 38.21	*A*	575.40 ± 38.21	*AB*	647.73 ± 66.18	*AB*
Belowground	356.79 ± 33.71	*A*	272.22 ± 47.68	*A*	313.65 ± 47.68	*A*	331.49 ± 82.58	*A*
Total Aboveground	1,128.31 ± 28.81	*B*	1,290.30 ± 40.74	*A*	1,207.03 ± 40.74	*AB*	1,287.24 ± 70.56	*AB*

The table lists the means for community productivity, measured for seventeen weeks. Aboveground and belowground biomass was partitioned at the end of the experiment. Total aboveground productivity represents the sum of clippings and aboveground biomass. Means and pooled standard errors are presented here by treatment. Different letters represent significantly different productivity levels, by partitioning, using a Tukey’s HSD test. Contrasting letters indicate significant differences in the means.

Where possible, grassland mesocosms were tested for species effects by treatment (S2 Fig). In polycultures containing *Poa annua var*. *reptans* and *P*. *supina*, mean standing biomass consisted of 17.4–87.4% and 38.8–58.0% of total aboveground biomass, respectively. In one 3-component polyculture treatment, *P*. *annua* produced 774.7 g•m^-2^ of biomass at harvest, which nearly equaled the most productive monoculture treatment (*P*. *supina*) that produced 785.8 g•m-^2^. The species *P*. *annua* was not one of the grasses selected at random to be grown in monoculture, therefore only its performance in polyculture is reported.

### Soil microbial diversity within multiple-species grasslands

**Fig 2:**
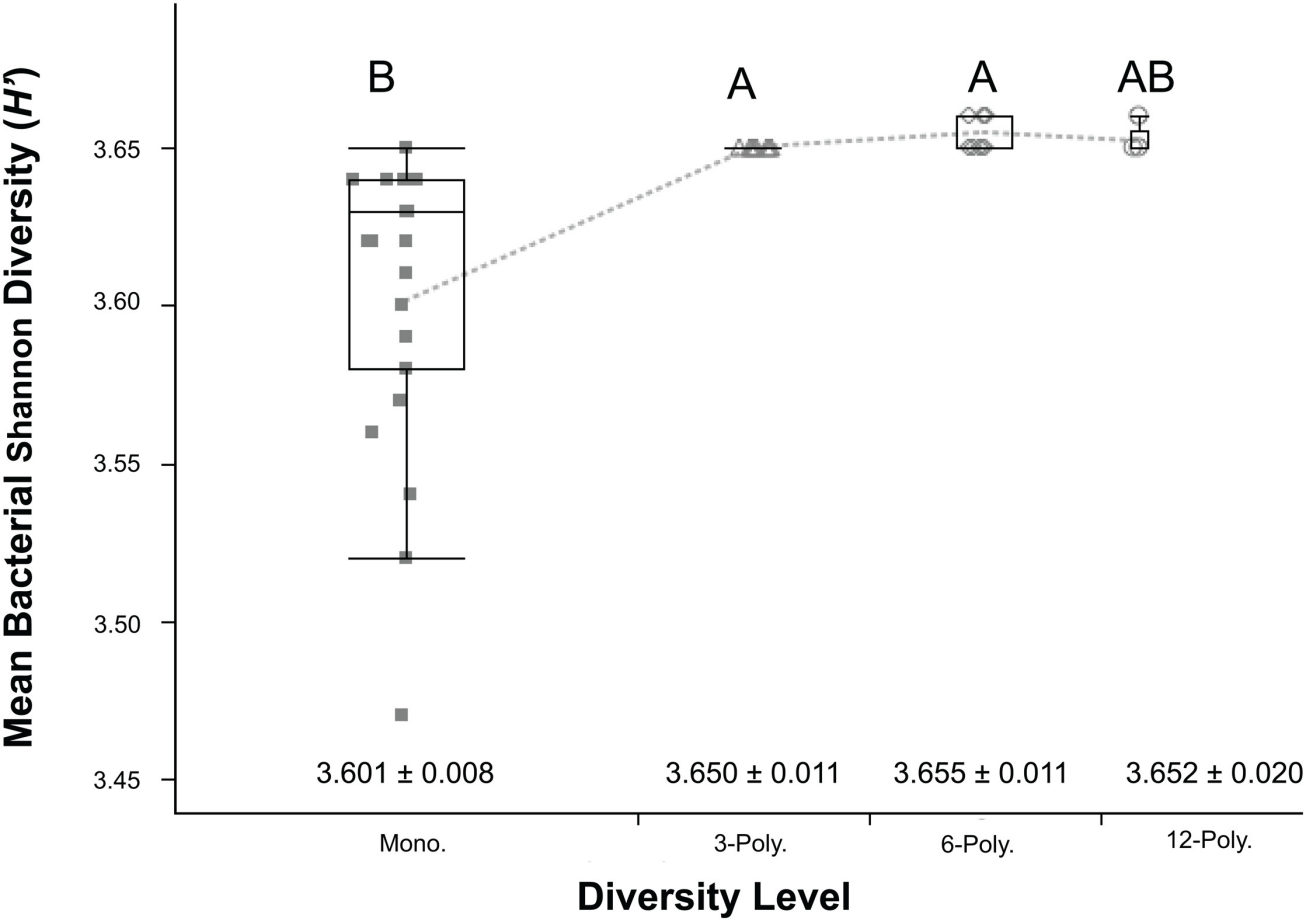
Soil bacterial richness, as measured by Shannon Diversity (*H’*) Index. The scores are displayed by grassland diversity levels: Monocultures or 1-way component (treatments 1–6), three 3-component polycultures (treatments 7–9), three 6-component polycultures (treatments 10–12), and a 12-component polyculture. Error bars represent ±1 SEM. Box plots with contrasting letters indicate significantly different means by Tukey’s HSD test.

**Fig 3:**
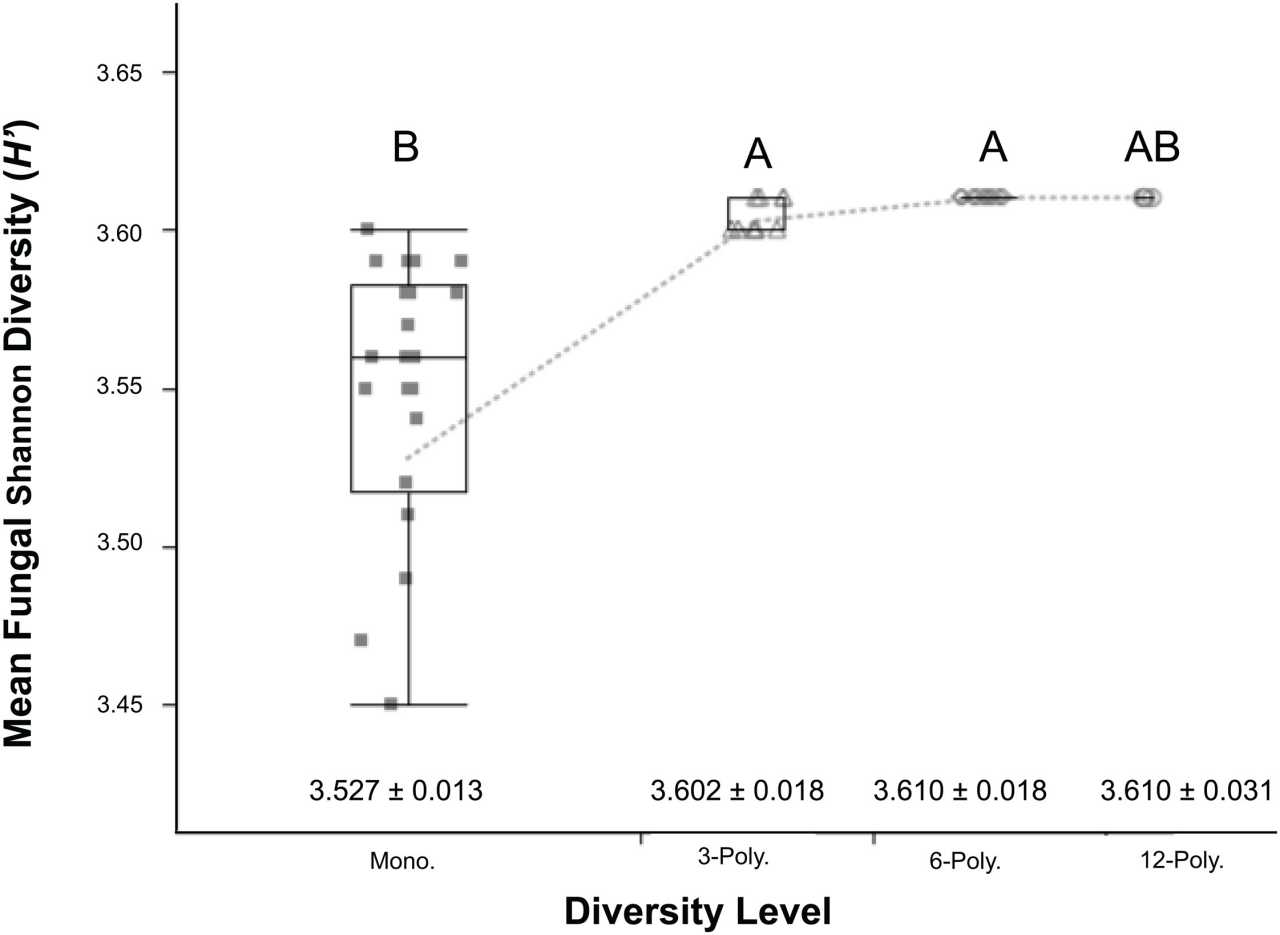
Soil fungal richness, as measured by Shannon Diversity (*H’*) Index. The scores are displayed by grassland diversity levels: Monocultures or 1-way component (treatments 1–6), three 3-component polycultures (treatments 7–9), three 6-component polycultures (treatments 10–12), and a 12-component polyculture. Error bars represent ±1 SEM. Box plots with contrasting letters indicate significantly different means by Tukey’s HSD test.

Shannon’s diversity (*H’*) for soil bacteria and fungi were calculated (Figs 2 and 3, respectively). Fungal relative abundance, as determined from peak florescence, was lower compared to bacterial samples. Fungal abundance was amplified one hundred-fold to resolve differences in community diversity. Bacterial communities in monoculture treatments displayed reduced richness compared to the polyculture treatments. Furthermore, the diversity of the 3- and 6-component polycultures were indistinguishable. No significant differences were found between the 12-component polyculture and the other polyculture treatments. As with bacterial community richness, fungal richness increased in response to greater plant diversity from one to multiple species. Differences in bacterial and fungal richness was difficult to detect with low statistical power at the highest diversity treatment (12-component, n = 5).

### Bacterial and fungal community structure with increasing plant diversity

**Fig 4:**
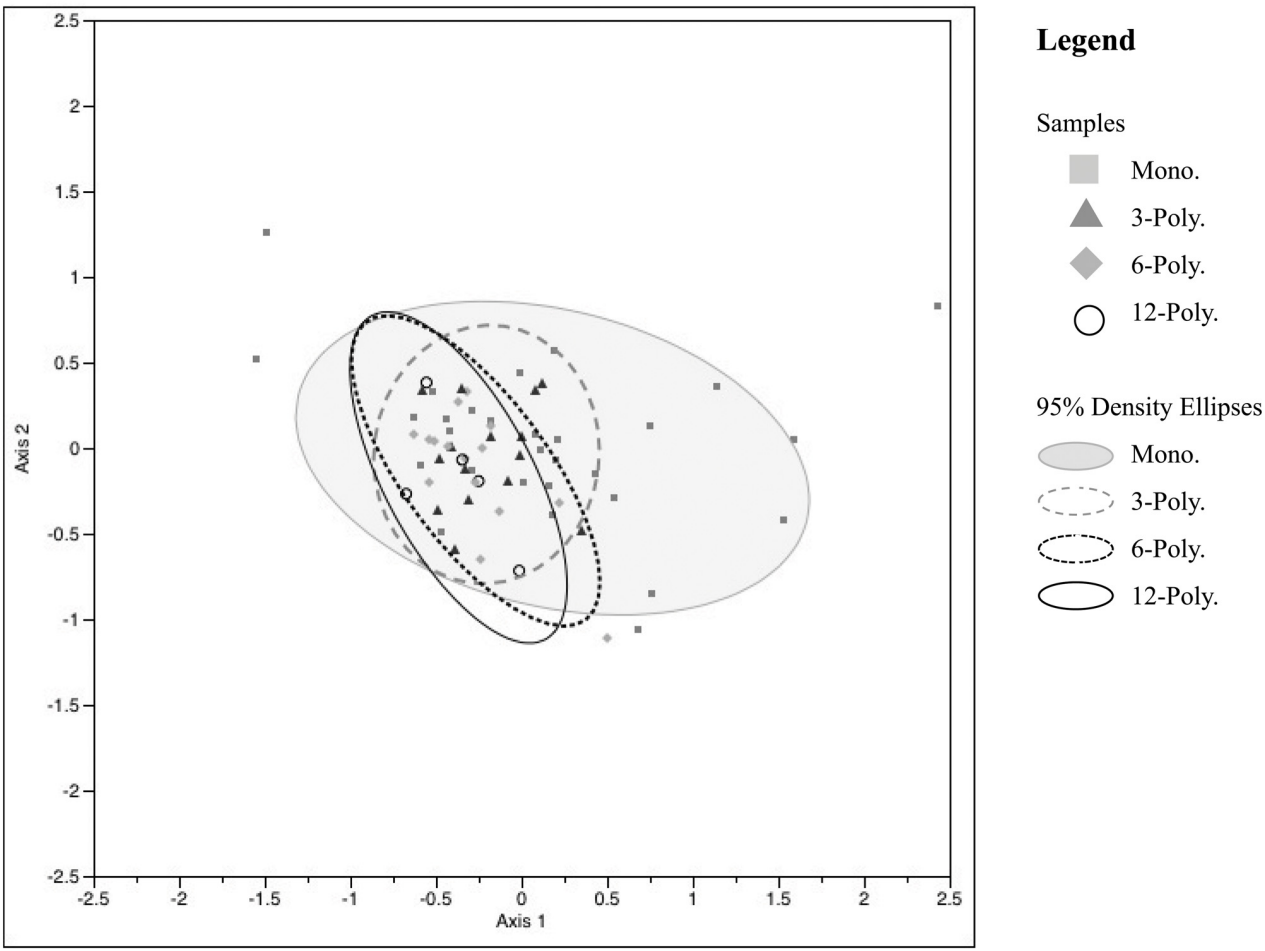
NMS ordination of bacterial community (T-RFLPs). Bacterial community structure is displayed by grassland diversity levels: Monocultures or 1-way component (treatments 1–6), three 3-component polycultures (treatments 7–9), three 6-component polycultures (treatments 10–12), and a 12-component polyculture. Axis 1 R^2^ = 0.888 Axis 2 R^2^ = 0.055, cumulative R^2^ = 0.943.

**Fig 5:**
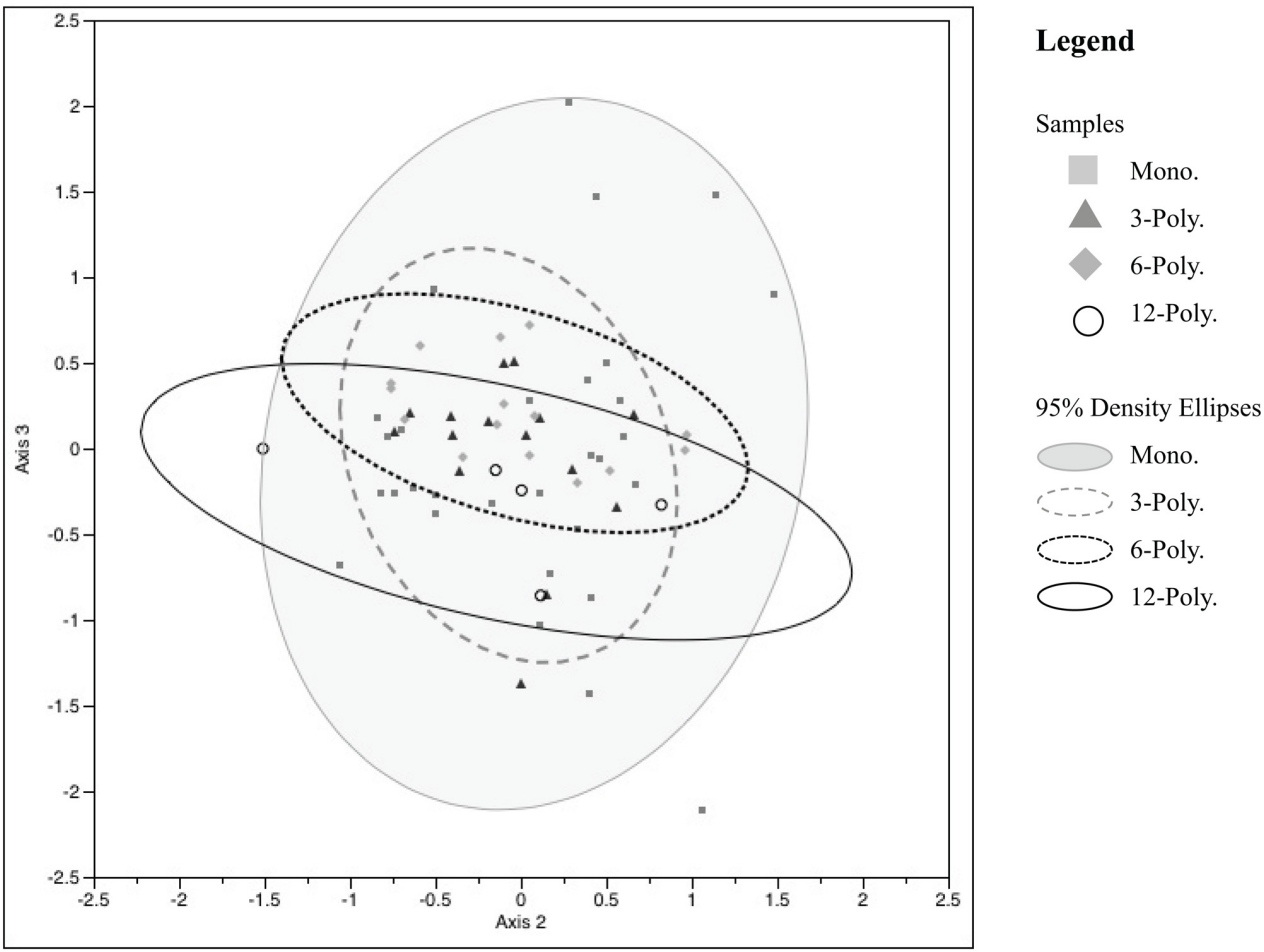
NMS ordination of fungal community (T-RFLPs). Fungal community structure is displayed by grassland diversity levels: Monocultures or 1-way component (treatments 1–6), three 3-component polycultures (treatments 7–9), three 6-component polycultures (treatments 10–12), and a 12-component polyculture. Axis 1 R^2^ = 0.245 (not shown), Axis 2 R^2^ = 0.257, Axis 3 R^2^ = 0.372, cumulative R^2^ = 0.875.

Soil bacterial and fungal communities in the urban grassland mesocosms were characterized using T-RFLP. NMS analysis of 199 bacterial TRFs using a Relative Sorensen distance measure resulted in the lowest stress model 9.27 and a 2-dimensional solution that accounted for 94.3% of the variability observed in samples. Soil bacterial communities under monoculture conditions show higher variability in community composition, however large portions of bacterial communities are shared regardless of diversity treatment. Variability in the community structures of the 6- and 12-part polycultures are similarly oriented (Fig 4). Fungal TRFs used a correlation distance measure for NMS analysis. Model stress was 14.09, producing a 3-dimensional solution accounting for 87.5% of the observed variation. NMS fungal data are presented in one biaxial plot (axes 2 and 3) which show the greatest differences in community variability (Fig 5). There is no underlying structure to the fungal communities in relation to diversity level treatments.

## Discussion

Researchers over the past 25 years have sought to understand the effects of plant species diversity on ecosystem processes. More recently, the theoretical concepts are being used in landscape design of urban ecosystems [40]. We applied the concepts of biodiversity and ecosystem function literature to the assemblage of urban grasslands using common plant species found in cool-season climates. Both nitrate leaching and aboveground plant productivity were modified when plant diversity increased in the experimental grassland mesocosms. These results confirm the potential to integrate ecological theory in the design of urban landscape features that include lawns, parks, and fields. Not only were ecosystem processes altered, but changes in ecosystem properties, specifically bacterial and fungal community structure, were observed. These results provide strong support for proceeding with *in situ* measurements of grassland diversity impacts on urban ecosystems.

Urban grasslands provide an important service in effectively retaining N in soils [41, 42], but no studies explicitly test the effect of turfgrass richness on N dynamics. In our study of grassland mixtures, diversity was negatively associated with NO_3_^-^ leaching. In comparison, root-zone extractable NO_3_^-^ across a native grassland diversity gradient was negatively correlated with diversity (R^2^ = 0.22), and reached a minimum between 8–12 species, beyond which NO_3_^-^ concentrations were not observed to be different Tilman [30]. Similarities between Tilman *et al*.*’s* findings and our study, both in terms of correlation strength and the amount of diversity needed to minimize NO_3_^-^ leaching, provide evidence that diversifying urban grasslands may have positive ecosystem services. Additionally, our findings concur with other studies that have found polyculture composition may be as important as species richness in determining ecosystem function [22, 33, 43, 44]. While individual species traits, interspecific competition, and complementarity should be considered, as has been suggested [45], the act of diversifying a plant community increases the likelihood of including a species that has a dominant effect on a measurable ecosystem trait.

In the case of N retention, NO_3_^-^ levels may be explained by enhanced plant uptake, incorporation into microbial biomass, gaseous loss (NH_3_, N_2_O, or N_2_), or a combination of these processes [32, 46]. This experiment was not designed for determining a complete N budget, however combustion analyses were performed to determine N content of aboveground standing biomass. Total tissue N (g) was not significantly different between diversity treatments (*P* = 0.08). Tissue N and productivity alone do not account for the increased NO_3_^-^ retained under higher diversity treatments. While belowground N content was not measured, soil microbiota are known to mediate the cycling of N soil pools through mineralization, N-fixation, and immobilization [46–48]. Microbial N immobilization may account for the observed NO_3_^-^ retention and should be included in future studies. Furthermore, some grass species, including perennial ryegrass, have been shown to produce nitrification inhibitors in the rhizosphere that may reduce nitrates available for leaching [49].

It is well known that plants associated with N-fixing bacteria can have different impacts on NO_3_^-^ leaching, compared to plants without such associations [33]. Two studies of species and functional group diversity effects in native grasslands found legumes were responsible for the greatest NO_3_^-^ leaching, and polycultures containing legumes leached more than non-legume containing mixtures [32, 33]. The higher rates of NO_3_^-^ leaching in legumes are consistent with our observations of *T*. *repens* ‘Microclover’ in the monoculture and polyculture treatments. While the high NO_3_^-^ leaching in legumes supports the concept of a single species dominating a measureable ecosystem trait, it also reinforces the value of diversity in limiting NO_3_^-^ leaching through complementarity and the additive effects of including species that abate the high NO_3_^-^ leaching values of a particular species.

Total aboveground productivity also showed increases from monoculture to polyculture treatments. Though there was a wide range in species-specific productivity, the results indicated that certain species were highly productive, even in polyculture. Interestingly, though *P*. *supina* and *P*. *annua* were highly dominant in monoculture or 3-polyculture, respectively; they were not as productive or dominant at higher diversity levels (Fig 2). Controlled manipulations of grassland diversity and composition have also concluded the identity of species present may be as important as functional richness in determining effects on ecosystem processes [33, 44, 50]. This finding supports the *sampling effect* hypothesis for describing biodiversity-productivity effects. The sampling effect proposes that more diverse communities have a greater likelihood of including a highly productive species; thus on average, a polyculture is more likely to be highly productive than a monoculture [45]. Despite not having all treatments in monoculture for comparison, biodiversity effects are apparent, even if differential sampling of the species pool is mechanistically driving observed diversity effects.

Belowground productivity was not correlated (R^2^ = 0.02) with plant diversity, which conflicts with BEF trends in native grasslands [22, 51]. This finding may be an artifact of sampling methods or experimental design. Mesocosm size was selected in order to facilitate high-diversity polycultures, yet container depth (18 cm) may have been insufficient to allow complete root-system development [52, 53]. Evidence of this was apparent upon destructive harvesting when a majority of samples were root-bound. Alternatively, root-sampling procedures could have resulted in a larger proportion of fine root loss.

In the context of urban grasslands, productivity is an important ecosystem property. High productivity is desired to quickly establish a closed turf stand to suppress invasion by weeds and pathogens [52, 53], and to grow at a sufficient rate to overcome maintenance-associated stresses (i.e. trafficking, use-related damage, and mowing). However, enhanced productivity also increases the frequency of mowing to maintain turf at a desired height. More frequent mowing has environmental impacts stemming from the burning of fossil fuels and economic consequences from labor, fuel, and repair costs [49]. This experiment suggests that on average, even moderate increases in diversity may only result in minor increases of productivity. While the contributions of all species included in this study were not individually measured, the collective response does underscore the role of plant species diversity in enhancing productivity.

Plant diversity had additional impacts on ecosystem properties that are important to note. Soil bacterial and fungal community structures were evaluated using PCR-based methods (T-RFLP profiling). Rhizosphere bacterial community composition was shown to be highly variable among monoculture treatments, resulting in different community compositions than observed in polyculture rhizospheres. Soil fungal communities were variable across treatments, and therefore, did not show distinguishable profiles by plant diversity treatments. Despite difficulties in discerning structural differences in rhizosphere microbial communities, our findings do provide evidence for a link between plant species and microbial community richness and evenness, as measured by the Shannon *H’* index.

While T-RFLP provides only a proxy of microbial diversity, based on fragments of PCR-based amplicons, it can give a reasonable estimate of changes to diversity and community structure across environmental variables [54]. Managed ecosystems tend to have lower diversity soil microbial communities when compared to natural environments [15, 37], thus we used *H’* as a reasonable diversity measure for this study. Our study found increasing plant diversity might have significant impacts on soil microbial richness. This finding concurs with results discussed in Kowalchuk [55], which found positive linkages between plant richness and soil microbial diversity. The authors noted that macrophyte diversity effects were limited to the rhizosphere, and bulk soil bacterial diversity was typically higher than the rhizosphere.

Changing land uses from unmanaged natural landscapes to managed urban or peri-urban grasslands can alter soil microbial communities and associated biogeochemical processes. Generally, reductions in soil microbial diversity are associated with high disturbance, managed ecosystems (e.g. agricultural and urban) [15]. Reductions in the heterogeneity of soil characteristics, specifically in microbial ammonia oxidizer diversity, were found under differing grassland irrigation and fertilization management regimes Webster [56]. In a short period of land use conversion, such as one year after a pine forest to turfgrass transition, shifts in microbial community structure were detected Yao [57]. Increasing urban grassland diversity may offer one means for offsetting potential negative consequences on soil microbial diversity. Maintaining diversity and complexity in soil microbial interactions is vital for soil health and function [58]. A suite of soil microorganisms are responsible for the cycling of C, N, and P from fixed to available forms, through an array of extracellular enzymes and metabolic processes [59, 60]. Reduced soil biotic diversity can negatively impact plant community structure and function [47]. Consequently, future studies should probe the strength of linkages between plant diversity and soil microbial community structure. Specifically, research should focus on differences in plant composition and traits on microbial community function in a variety of landscape designs.

## Conclusions

Urban grasslands have traditionally reduced the diversity of the native or agricultural landscapes, which they replace. Such reductions in species richness have been known to have negative consequences for ecosystem functioning. As we have shown, plant diversity enhances ecosystem function, specifically NPP, N-retention, and soil microbial community diversity in experimental urban grassland assemblages. More over, we provide evidence that ecological theory derived from natural ecosystems is applicable to urban ecosystems. Where possible, BEF and other ecological theories, should be applied to the design and management of developed landscapes to explicitly enhance ecosystem services. If results from this study are confirmed in the field, it suggests that grassland biodiversity can be manipulated to assist in N management, reducing contributions toward the eutrophication of urban watersheds. More research is required to elucidate if positive biodiversity-nutrient retention effects occur when urban grassland communities experience typical *in situ* stresses.

## Supporting Information

S1 Fig Leachate NO_3_^-^ (mg N ∙ L^-1^) concentration from mesocosms. Individual treatments mean leachate NO_3_^-^ concentrations with pooled error bars representing ±1 SEM. The grand mean for all treatments is the solid horizontal line, while the dashed horizontal line is the mean of the monoculture treatments.
(EPS)

S2 Fig Partitioning of the standing aboveground biomass by visual sorting of community components. The legend lists each individual component and its composition across the assemblages: Monocultures or 1-way component (treatments 1–6), three 3-component polycultures (treatments 7–9), three 6-component polycultures (treatments 10–12), and a 12-component polyculture. Legend abbreviations can be found in Table 1, with the addition of PoolTF = pooled tall fescue; PoolFF = pooled fine fescue; and PoolRG = pooled ryegrass for genotypes pooled together that could not be visually separated.
(EPS)
